# Dissipation Dynamic, Residue Distribution and Risk Assessment of Emamectin Benzoate in Longan by High-Performance Liquid Chromatography with Fluorescence Detection

**DOI:** 10.3390/molecules28083346

**Published:** 2023-04-10

**Authors:** Yanping Liu, Haibin Sun, Xiaonan Wang, Hong Chang, Siwei Wang

**Affiliations:** Institute of Plant Protection, Guangdong Academy of Agricultural Sciences, Key Laboratory of Green Prevention and Control on Fruits and Vegetables in South China Ministry of Agriculture and Rural Affairs, Guangdong Provincial Key Laboratory of High Technology for Plant Protection, Guangzhou 510640, China

**Keywords:** emamectin benzoate, longan, dissipation dynamic, terminal residue, residue distribution, risk assessment

## Abstract

A derivatization method combined with high-performance liquid chromatography–fluorescence detection (HPLC–FLD) was used to evaluate the dissipation, residue distribution and risk assessment of emamectin benzoate in whole longan and pulp. The average recoveries were 82–111% with relative standard deviation (RSD) less than 11%. The limit of quantification (LOQ) was 0.001 mg/kg in longan and pulp. The half-lives were 3.3–4.2 days. The terminal residues in whole longan were <0.001–0.025 mg/kg applied two and three times at two levels of dosage with PHIs of 10, 14, and 21 days. The residues in whole longan had a higher quantity than those in the pulp, and the terminal residues of pulp were all lower than LOQ (0.001 mg/kg). The chronic risk of emamectin benzoate was not negligible to humans depending on ADI% value, which was higher than 1; and the acute risk was acceptable to the consumer. This study could provide guidance for the safe use of emamectin benzoate in longan and serve as a reference for the establishment of maximum residue limits (MRLs) in China.

## 1. Introduction

Longan (*Dimocarpus longan* Lour.) is a kind of tropical and subtropical fruit originating in China. Due to the nutrients, including sugars, organic acids, and amino acids as well as abundant functional components containing polyphenols, flavonoids, alkaloids, polysaccharides, vitamins, nucleotides (or nucleosides), tannins, proanthocyanidins, and other bioactive compounds, longan pulp has been used as a traditional Chinese medicine for a long history to promote blood metabolism, soothe nerves, relieve insomnia, prevent amnesia, extend longevity, cure neural pain and swelling, treat palpitation and serve as anti-hyperglycemic agents in Asian countries [1,2,3]. Longan is an agricultural product for both food and medicine. Polysaccharides, which are excellent acetylcholinesterase inhibitors, have many pharmacological activities such as immune regulation, anti-inflammatory, antioxidant, anti-fatigue, and anti-tumor properties, and regulating the cholinergic system is one of the mechanisms of longan pulp to improve memory impairment. Phenolic substances in longan pulp mainly exist in free form, and their content is 16.92~109.24 mg/100 g. The antioxidant capacity of phenolic substances in longan pulp is significantly positively correlated with the content of phenolic substances. Longan pulp contains malic acid, tartaric acid, oxalic acid, succinic acid, citric acid and other organic acids, 17 amino acids and 28 volatile components [4]. As an edible fruit and traditional Chinese medicine, longan has been consumed for thousands of years. China is the main producer and has the main global cultivation area and output [5]. However, longan is vulnerable to a wide range of pests and diseases. The main diseases include witches’ brooms, peronophythora litchi, anthracnose, and gray speck disease. *Conopomorpha litchiella*, *Conopomorpha sinensis*, *Cornegenapsylla sinica*, *Tessaratoma papillosa*, *C. litchiella*, and *T. papillosa* were identified as major insect pests, causing visible external damage [6]. Great efforts have been made to control the damage and to maintain quality of longan [7]. At present, more than 80% of longan orchards still rely mainly on chemical control. Only a few commonly used pesticides, such as chlorpyrifos, cypermethrin, and prochloraz, are used. Pesticides are a common means of pest control and play an important role in ensuring the quality and yield of crops. However, most pesticides are toxic to a certain extent, and non-standard use of pesticides may lead to pesticide residues exceeding the standard, bringing potential health risks to consumer groups. Therefore, we should focus on the pesticide residues in crops. Monitoring of pesticide residues in longan is currently indispensable to ensuring food safety and protecting consumers against potential health risks.

**Figure 1:**
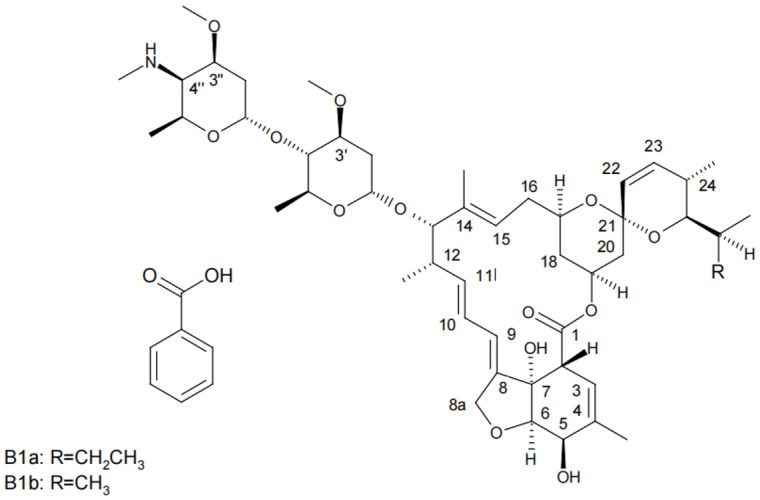
Chemical structures of emamectin benzoate (R = CH_2_CH_3_ for emamectin B1a benzoate; R = CH_3_ for emamectin B1b benzoate).

A macrocyclic lactone insecticide designated emamectin benzoate is developed from the avermectin that *Streptomyces avermitilis* produces [8]. There is a minimum of 90% (4′′R)-4′′-deoxy-4′′-(methylamino) avermectin B1a benzoate and a maximum of 10% (4′′R) in the combination compounds of 4-deoxy-4-(methylamino) avermectin B1b [9]. Insects are paralyzed within hours of consumption and expire 2–4 days afterward due to the inhibiting neurotransmitter γ-aminobutyric acid being stimulated by emamectin benzoate [10]. Many countries have approved its use in cereals, vegetables, fruits, and other crops. In 2011, the Joint FAO/WHO Meeting on Pesticide Residues reviewed emamectin for the first time. Emamectin B1a benzoate is the residue defined for MRL conformity or dietary intake measurement for animal and plant commodities [11]. Figure 1 illustrates the composition of emamectin benzoate. 

Pesticide residue measurement processes usually include instrumental analysis and sample pretreatment. To achieve precise measurement, optimal cleanup procedures are crucial for preventing instrument contamination and matrix interference effects. Consequently, developing a highly sensitive technique to identify pesticide residues in longans is essential to mitigate health risks and encourage worldwide trade. Pretreatment of samples is essential when assessing the precision and dependability of pesticide residue detection. Solid-phase microextraction (SPME) is the most typical pretreatment procedure used for foods [12], with other examples including solid-phase extraction (SPE) [13]; matrix solid-phase dispersion (MSPD) [14]; the quick, easy, cheap, effective, rugged, and safe (QuEChERS) method [15]; and derivatization. Among them, the QuEChERS method is widely used for sample preparation of cereals and fruits. It is a simple, stable, eco-friendly, low-cost, and efficient method. However, the limit of quantification (LOQ) of emamectin benzoate was only 0.01 mg/kg when applying the QuEChERS method and 0.001 mg/kg when using derivatization. Hence, derivatization is an important pretreatment method to improve emamectin benzoate sensitivity. The most commonly used methods to detect pesticide residues in different matrices are high-performance liquid chromatography–tandem mass spectrometry (HPLC-MS/MS) techniques, high-performance liquid chromatography (HPLC), gas chromatography (GC), and gas chromatography–mass spectrometry (GC-MS). Among them, HPLC-MS/MS has emerged as an effective means of detecting pesticide residues owing to the high resolution, selectivity, and power of this approach, enabling the detection of most pesticides of interest in a range of foodstuffs. HPLC is a commonly used detection method, the main reason for which is its low price and wide range of detection. However, no studies have been carried out on emamectin benzoate method validation in longan matrices and their residual properties in longan. 

The Bla of emamectin benzoate has been used to formulate the maximum residue limit (MRL) by the Joint Meeting on Pesticide Residues (JMPR). The Codex Alimentarius Commission (CAC), the United States, Japan, and China, have not formulated a maximum residue limit (MRL) of emamectin benzoate on longan. The MRL values are specific regulations to control the levels of pesticide residues found in foods. It is anticipated that pesticide residue levels higher than the defined MRL standards could be dangerous to animals and humans. Moreover, a risk evaluation of food pesticide residues can provide an empirical foundation for safety risk management, application advice, quality oversight, and MRL. However, the assessment of the dietary risk presented by pesticide residues in Chinese longan is the subject of few investigations. Therefore, we established and validated a technique for the detection of emamectin benzoate on longan using derivatization coupled with HPLC. The established method was applied to detect the pesticides on real longan samples, and the results were used to assess the dietary intake risk levels for Chinese consumers.

As far as we know, some studies have analyzed the emamectin benzoate residue in agricultural products, such as rice [16], okra [17], tomato [18], vegetables [19,20], cotton [21], tea [22], and tobacco [23]. There are few reports about the dissipation and residue of emamectin benzoate in apple [24], cabbage [25,26], cowpea pods [27], and paddy [28]. The instruments used in the above methods included HPLC-FLD, HPLC, and HPLC-MS/MS. The main pretreatment methods were derivatization, SPE, the QuEChERS method, and dispersive solid-phase extraction (DSPE). SPE is a tedious and time-consuming method involving the use of a large number of organic solvents and nitrogen blowing, which is relatively cumbersome; however, SPE has the advantage of a lower matrix effect and good purification effect. DSPE and the QuEChERS method can achieve better purification with the addition of a small amount of adsorbent to the extraction solution to adsorb impurities, which is generally used in conjunction with HPLC-MS/MS. The detection limit of emamectin benzoate was 0.0001 mg kg^−1^ in rice, 0.01 mg kg^−1^ in cowpea pods, 0.001–0.01 mg kg^−1^ in cabbage, 0.05 mg kg^−1^ in okra, 0.01 mg kg^−1^ in tomato, 0.0001 mg kg^−1^ in mushroom, 0.00012–0.00021 mg kg^−1^ in vegetables, and 3.2 ng kg^−1^ in tobacco, respectively. However, no work has been carried out to estimate the dissipation behavior and terminal residues of emamectin benzoate in longan under open-field conditions.

The maximum residue limit (MRL) of emamectin benzoate in longan has not been legislated in China, and no work has been carried out to determine the emamectin benzoate residue in longan. The present study aims (1) to establish an effective and sensitive method for emamectin benzoate based on the HPLC-FLD technique in longan samples; (2) to evaluate the dissipation kinetics and terminal residues of emamectin benzoate in longan under field conditions, distribution in whole longan, and pulp; and (3) to assess the safe use of emamectin benzoate in longan and to assess the risk assessment of emamectin benzoate based on the results of the terminal residue. 

## 2. Results and Discussion

### 2.1. Method Validation

The parameters of recovery, accuracy, and precision were used to validate the analytical technique. Recoveries were calculated to assess the technique’s precision as well as accuracy. Linearity was determined using matrix-matched samples in the 0.001–2 mg/L range for whole longan and pulp in the present study. The r^2^ association coefficients were greater than 0.9980 in both cases. Table 1 shows the precision and accuracy derived from recovery analyses of five replicate samples (*n* = 5) at three levels of emamectin benzoate. 

**Figure 2:**
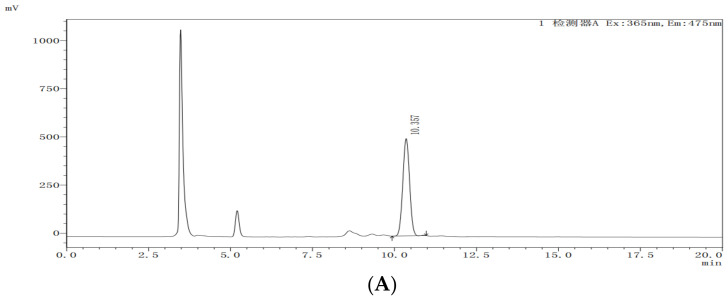

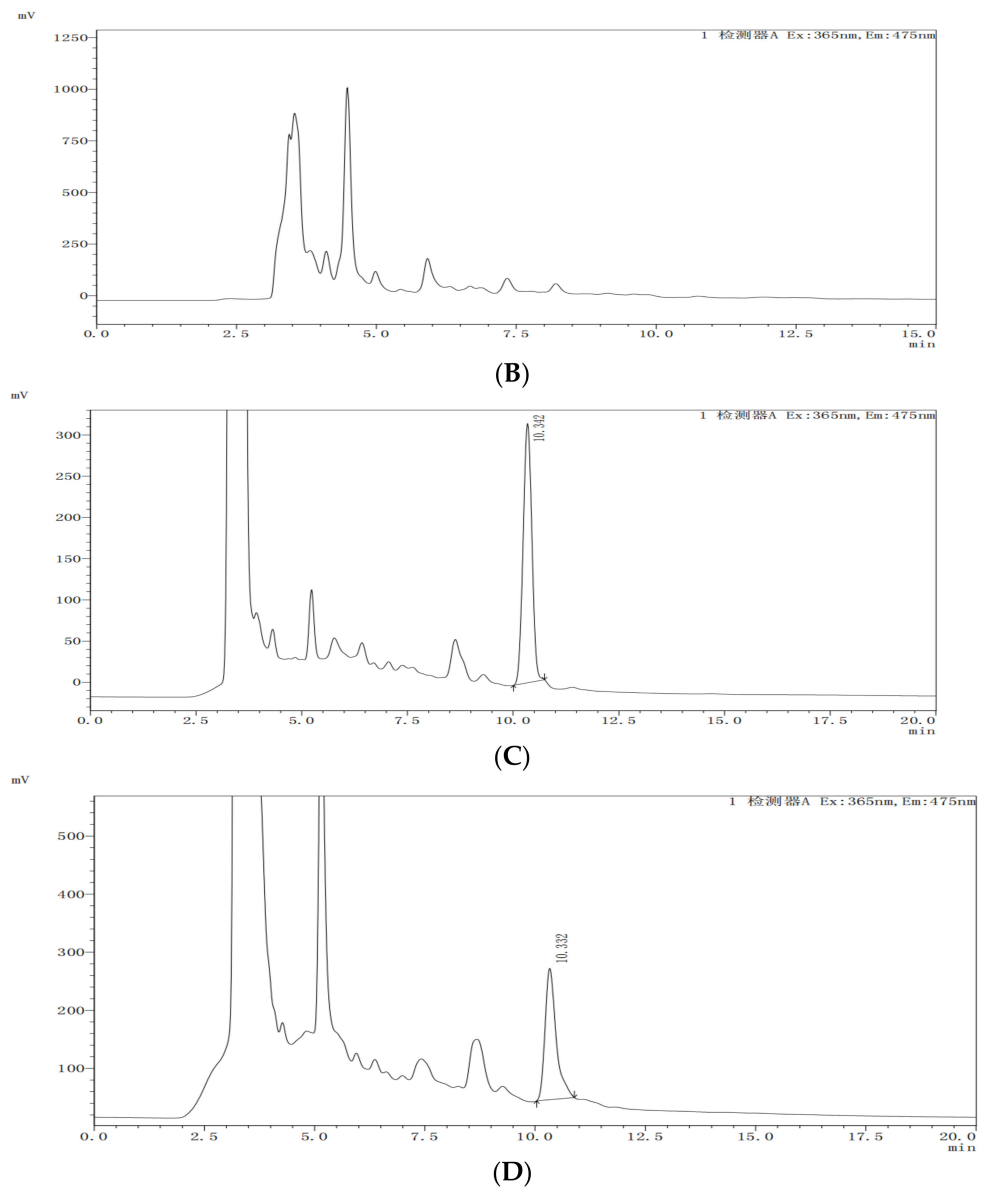
Chromatogram of emamectin benzoate standard, blank, spiked sample, and real sample (Lateral axis is time, and longitudinal axis is response intensity). (**A**) emamectin benzoate standard (1 mg/kg); (**B**) longan CK; (**C**) spiked longan sample (0.1 mg/kg); (**D**) longan real sample. Note: The Chinese letters in this figure mean Detector.

For all levels of concentration, the mean recoveries were satisfactory, ranging from 99–111% with RSDs of 5–11% in whole longan and 82–83% with RSDs of 4–5% in pulp, respectively, which illustrated that the proposed method meets typical validation requirements for pesticide residue analysis. LOQs were defined as the minimum fortified concentrations of analyte in matrix with a signal-to-noise ratio of 10, which was 0.001 mg/kg for both whole longan and pulp, so the LOQ of emamectin benzoate in whole longan and pulp were 0.001 mg/kg. The results indicated the method within the acceptable range had good accuracy and repeatability. The representative HPLC-FLD chromatograms for blank and spiked samples are shown in Figure 2.

### 2.2. Dissipation of Emamectin Benzoate in Longan

**Figure 3:**
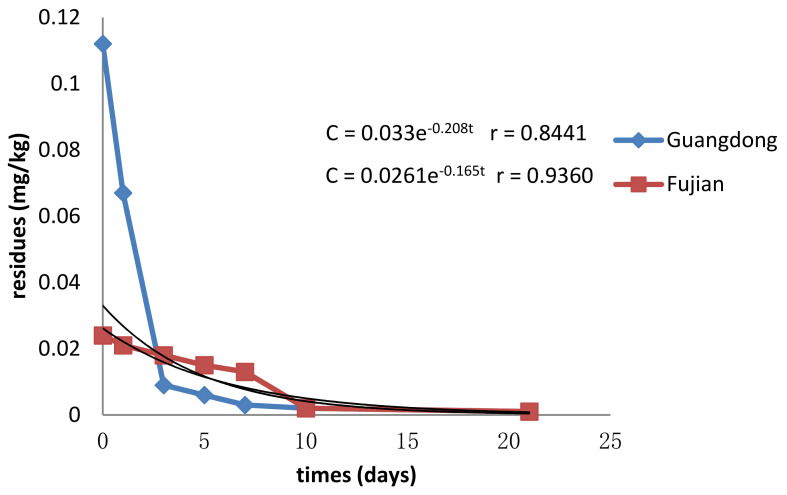
The dissipation pattern of emamectin benzoate in longan (lateral axis is collected sample time, and longitudinal axis is residues).

Figure 3 depicts the dissipation pattern of emamectin benzoate in longan under open-field conditions. Emamectin benzoate was initially deposited at 0.112 mg/kg in the Guangdong region and 0.024 mg/kg in the Fujian region, according to the findings, which showed that longan in the Guangdong region acquired a higher concentration of the compound than those in the Fujian province. From 0 to 3 days after application, the concentrations of emamectin benzoate in the Guangdong treatment dropped considerably in longan, achieving 0.009 mg/kg with a 91.82% of high loss. The impact of physical and chemical factors, such as temperature, moisture, sunlight, pH, etc., may be attributed to dissipation in emamectin benzoate residues. Furthermore, some studies suggest that the development dilution factor may influence residue reduction [29]. The above finding indicated that harvesting was effective and safe 14 days after administering the prescribed amount of emamectin benzoate.

Dissipation in the residues of emamectin benzoate may be attributed to the effect of some physical and chemical factors such as temperature, moisture, sunlight, and pH. In addition, some researchers reported that growth dilution factor also might play a significant role in residue decline [30]. This result suggested that it is safe to harvest 14 days after applying the recommended dose of emamectin benzoate. 

### 2.3. Terminal Residues of Emamectin Benzoate in Longan

Table 2 summarized the terminal residue of emamectin benzoate in longan. When emamectin benzoate was applied 2–3 times at 10 mg/kg application dosage, the residues of whole longan were 0.001–0.012 mg/kg, <LOQ–0.005 mg/kg, and <LOQ–0.001 mg/kg based on PHIs of 10, 14, and 21 days.When application was 15 mg/kg (the PHI and times was the same as above), the residues were 0.002–0.025, 0.001–0.012, and <LOQ–0.003 mg/kg, respectively. It was easily observed that shorter PHI, higher application, and more application times had a greater concentration of terminal residues. 

The residues in whole longan had a higher quantity than those in the pulp, and the terminal residues of pulp were all lower than the LOQ (0.001 mg/kg). It could be predicted that most of the residues were concentrated in the peel. Due to emamectin benzoate ME being applied to the peel, the pulp was not directly exposed to the pesticide. On the other hand, emamectin benzoate is a fat-soluble insecticide with its Kow logP (5.0, pH 7) (The e-pesticide manual, Version 3.0) making it more likely to absorb in the peel. Hence, only a very small quantity of residues permeated into the pulp. 

From the results, the conclusion could be drawn that the residues of emamectin benzoate in longan increased with application dosage, but the differences in the residues were not significant at the six regions. The harvest time substantially affected the terminal residues. After 21 days of application, the residues of emamectin benzoate in all of the longan samples were no more than 0.001 and 0.003 mg/kg at the recommended dosage and 1.5 times the recommended dosage, respectively. The fact was that the terminal residue level was higher in shorter PHI. 

### 2.4. Risk Assessment

Based on the common food (fruit) utilization in China, a risk assessment for emamectin benzoate in longan has previously been investigated [29]. Chronic and acute exposure evaluation are components of dietary exposure assessment, a crucial stage in the risk evaluation process [31]. The ADI% and ARfD% were used to calculate the amount of emamectin benzoate ingested from consuming longans (Seen in Table 3). Emamectin benzoate residues in pulp samples were subjected to risk evaluation using ADI values (0.0005 mg/kg, bw) and ARfD (0.02 mg/kg, bw) obtained from the JMPR study [32]. The outcomes of consumption estimates that fell below the LOQ were used as LOQ values. The STMR of emamectin benzoate in pulp were all 0.001 mg/kg at 14 days (21 days) of PHI, respectively.

Table 3 exhibited the short-term and long-term risk assessment of emamectin benzoate in the whole longan. The ADI% values were 130.1%, which meant that there was unacceptable chronic risk with the exposure to the pesticides via longan consumption. From a public health point of view, these results indicated that the observed levels of emamectin benzoate residues in longan may cause chronic health risk to consumers. The reason for this phenomenon is the ADI value of emamectin benzoate is very low (0.0005 mg/kg bw). Secondly, the dietary risk mainly comes from the large number of light vegetables, and the MRL of cabbage is 0.1 mg/kg. Finally, because the dietary intake of fruit was low, and the residues in longan pulp was very low, the proportion of fruit intake should actually be very low. For risks exceeding 100%, China refers to the evaluation results of JMPR to establish an MRL value. The residues of emamectin benzoate in pulp were all below the LOQ, so the HR was 0, and the ARfD% was 0, which showed a negligible acute risk. 

## 3. Material and Methods

### 3.1. Chemicals and Reagents

Emamectin benzoate (95.5%) standard was purchased from Chem Service (West Chester, PA). A microemulsion (ME) containing 1% emamectin benzoate was obtained from Hebei Boken Agricultural Co., Ltd. (Hebei, China). HPLC-grade acetonitrile and methanol were purchased from Thermo Fisher Co., Ltd. (Pittsburgh, PA, USA). Analytical grade activated anhydrous sodium sulfate and sodium chloride were obtained from Sigma-Aldrich (Steinheim, Germany). N-methylimidazole and trifluoroacetic anhydride were purchased from Agela Technologies Company (Tianjin, China). The water used was purified with a Simplicity UV water-purification system from Millipore (Bedford, MA, USA). 

Individual standard pesticide stock solutions (1000 mg L^−1^) were prepared in acetonitrile and stored at −20 °C. The working standard solutions required for fortification and calibration were freshly prepared from stock solution by dilution with acetonitrile. The solvent calibration solutions were prepared by diluting the stock solutions with acetonitrile; similarly, the matrix-matched ones were achieved by diluting the stock solutions with blank extracts of all matrices. All standard solutions were stored at 4 °C before use.

### 3.2. Field Trials and Sample Preparation

The field experiments, including the residue dynamic experiments and terminal residue experiments, were carried out in six sites (Guangdong Province (Guangzhou City and Maomign City), Guangxi Province (Fangchenggang City), Yun’nan Province (Baoshan City), Fujian Province (Zhangzhou City), and Hainan Province (Haikou City)) in the year 2017 according to the “Guideline on Pesticide Residue Trials” issued by the Ministry of Agriculture, the People’s Republic of China (NY/T788-2004), and the recommendations of the pesticide labels. 

The terminal residue experiment at the supervised field trial was carried out with the recommended dosage of 10 mg/kg and a higher dosage of 15 mg/kg (1.5 times the recommended dosage). A 1% emamectin benzoate microemulsion (ME) formulation was sprayed two and three times with an interval of 7 days between each application at both low and high levels. Each plot contained two longan trees. Each treatment was composed of three replicate plots and one control plot. Representative longan samples (2 kg) were randomly collected at preharvest intervals (PHIs) of 7, 14, and 21 days from several points in each plot after the last spraying. The terminal residue experiments samples included the whole longan fruit and longan pulp. The longan samples collected were divided into small amounts using quartering before being homogenized for analysis. 

In order to investigate the dissipation of emamectin benzoate in longan, 1% emamectin benzoate microemulsion (ME) dissolved in water was sprayed to longan plots at a dosage of 100 mg/kg when the longan reached 50% of the size of ripe longan. A plot of the same size but no emamectin benzoate application was compared simultaneously. Representative longan samples were randomly collected from each plot to evaluate dissipation 2 h, 1, 3, 7, 10, 14, and 21 days after application. All of the samples were stored at −20 °C for further analysis.

2.5 kg longan samples were provided, and then were homogenized immediately without being washed or peeled. Placed the homogenized samples into bottles, and frozen at −20 °C. The frozen samples needed to be transported to laboratory as eraly as possible, and always kept frozen until analysis. 

### 3.3. Extraction and Derivatization Procedures

The sample (20 g) of whole longan or pulp was weighed into a 50 mL centrifuge tube, and 40 mL of acetonitrile was added and vigorously shaken for 1 min using a vortex mixer at a speed of 15,000 rpm/min. After addition of 5 g NaCl, the samples were shaken vigorously for 1 min and centrifuged for 5 min at 3800 rpm. An aliquot of 20 mL supernatant was evaporated by using rotary vacuum evaporator at 40 °C to dryness and the extract was redissolved in 1 mL acetonitrile for derivatization.

Next, 100 µL derivatization agent A (N-methylimidazole:acetonitrile = 1:1 (*v/v*)) was added and shaken for 1 min. After 150 µL derivatization agent B (trifluoroacetic anhydride: acetonitrile = 1:2 (*v/v*)) was added, the tubes were capped immediately and the contents vortexed for 1 min. Methanol was added to mix evenly and allowed to stand for 15 min. The sample was transferred into a vial for instrumental analysis after filtration through a 0.45 μm polypropylene filter membrane. 

### 3.4. Instrumentation and HPLC Analytical Conditions

Emamectin benzoate was determined using a Shimadzu LC 20A HPLC (Shimadzu, Kyoto, Japan) with a fluorescence detector, operated at an excitation and emission wavelength of 365 and 470 nm, respectively, with the Thermo Betabasic-18 (250 mm × 4.6 mm, 5 μm) column (Thermo Fisher Scientific, Waltham, MA USA). The column oven temperature was set to 25 °C. The flow rate was 0.8 mL/min. The mobile phase consisted of acetonitrile (A) and water (B), and A/B = 90/10 (*v/v*). The injection volume was 10 μL. Under the above conditions, the emamectin benzoate time was approximately 10.4 min (total run time = 15 min). 

### 3.5. Methodological Validation

Values for the linear equation, the limit of quantification (LOQ), and the recovery rate were used to evaluate the precision and dependability of the designed methodology. Linear findings for prepared standards were observed within the 0.001–2 mg L^−1^ range. With five repetitions per concentration, recovery rate values were evaluated using blank food samples spiked with emamectin benzoate at concentrations of 0.001, 0.01, or 0.1 mg kg^−1^. To evaluate the methodological accuracy, relative standard deviations (RSDs) from this study were used. The LOQ of each compound was regarded as the lowest observed spiked quantity in the determined matrix (SANTE/11813/2017).

### 3.6. Statistical Analysis

Dissipation dynamics are calculated based on first-order kinetic equations C_t_ = C_0_e^−kt^, where C_t_ represents the emamectin benzoate residue at the different time t, C_0_ represents the original residues after emamectin benzoate application, and k is the dissipation constant. Calculating the half-lives (t_1/2_) of emamectin benzoate using this formula, ln2/k. The calculation of degradation rate is to subtract the residual amount at different time periods from the initial residue, and then divide it by the initial residue.

Based on the common food (fruit) utilization in China, a risk assessment for emamectin benzoate in longan has been investigated. Chronic and acute exposure evaluation are components of dietary exposure assessment, a crucial stage in the risk evaluation process [33]. ADI% was the chronic risk quotient, which could be calculated from the following formula:

ADI% = (STMR × FI)/(ADI × bw) × 100%

where STMR is an abbreviation for supervised trials median residues, the unit is mg/kg; FI is an abbreviation for food intake, and the unit is kg/day; ADI is an abbreviation of acceptable daily intake, the ADI values of emamectin benzoate was 0.0005 mg/kg, bw obtained from the JMPR study; and bw (kg) stands for the mean body weight (the bw is 63 kg for a Chinese adult). 

ARfD% is the acute risk quotient, which is estimated consumer health risk by intaking food in the short term, was calculated on the basis of the HR and the acute reference dose (ARfD, mg/kg/d), expressed as the following equation:

ARfD% = (U × LP × *v* +(LP − U) × HR)/(ARfD × bw) × 100%

where ARfD is an abbreviation for acute reference dose (the ARfD of emamectin benzoate was 0.02 mg/kg, bw); U(kg) represents the weight of the edible portion of a single longan; LP (kg) is an abbreviation for large portion; *v* is the variability factor (according to the recommend by GEMs/Food, *v* = 3); HR (mg/kg) represents the highest emamectin benzoate residue levels in longan (the undetected sample is assigned a value of 0). 

## 4. Conclusions

In conjunction with an HPLC-FLD, the derivatization technique could be used to identify the quantity of emamectin benzoate residue in the longan and pulp. To ensure the safety and suitable utilization of emamectin benzoate, the risk evaluation, distribution, terminal residue, and dissipation of the emamectin benzoate were examined. Emamectin benzoate degraded immediately, according to the observations, with a half-life of 3.3–4.2 days in longan, accompanied by the first-order kinetics rule. The pulp contained residues that were all lower than the LOQ (0.001 mg/kg), the whole longan terminal residues had a significantly higher quantity than those in the pulp. In consumers, the danger posed by emamectin benzoate at the recommended dosage presented a non-negligible threat based on the value of the chronic risk, which was greater than 1. The principle of dietary risk assessment in China is to maximize risk. Risk assessment is required for all crops registered for this pesticide, hence, the actual risk of emamectin benzoate may not be so high. In summary, the current research could be used as a guide for the appropriate application of emamectin benzoate in longans. The findings could contribute to introducing a practical maximum residue level (MRL) for emamectin benzoate in longans.

## Figures and Tables

**Table 1:** Performance characteristics of the method for emamectin benzoate in the whole longan and pulp.

Matrix	Fortified Level (mg/kg)	Average Recovery (%, *n* = 5)	RSD (%)	Correlation Coefficient	LOQ (mg/kg)
longan	0.001	99	11	0.9992	0.001
	0.01	99	8		
	0.1	111	5		
pulp	0.001	83	4	0.9987	0.001
	0.01	82	5		
	0.1	82	4		

**Table 2:** Terminal residues of emamectin benzoate in whole longan.

Dosage (mg/kg)	Spray Times	Days after Treatment (day)	Residues in Whole Longan (mg/kg)					
			Guangzhou	Maoming	Baoshan	Fangchenggang	Zhangzhou	Haikou
10	2	10/14/21	0.007/0.003/<LOQ	0.009/0.005/<LOQ	0.010/0.003/<LOQ	0.005/0.002/0.001	0.004/0.001/<LOQ	0.003/0.001/0.001
	3	10/14/21	0.009/0.004/<LOQ	0.012/0.005/<LOQ	0.012/0.004/<LOQ	0.006/0.002/0.001	0.005/0.001/<LOQ	0.003/0.002/0.001
15	2	10/14/21	0.009/0.006/0.001	0.017/0.007/0.001	0.018/0.008/0.003	0.008/0.003/0.001	0.005/0.002/0.001	0.004/0.002/<LOQ
	3	10/14/21	0.011/0.007/0.001	0.025/0.012/0.001	0.021/0.009/0.003	0.012/0.004/0.001	0.008/0.002/0.001	0.017/0.007/<LOQ

**Table 3:** The risk evaluation of emamectin benzoate in longan.

Food Classification	Fi (kg)	Reference Residue Limits (mg/kg)	Sources	NEDI (mg)	ADI (mg/kg)	Risk Quotient (%)
Rice and its products	0.2399	0.02	China	0.004798	0.0005 × 63	
Flour and its products	0.1385					
Other cereals	0.0233	0.05	China	0.001165		
Tubers	0.0495					
Dried beans and their products	0.016	0.05	China	0.0008		
Dark vegetables	0.0915	0.1	China	0.00915		
Light vegetables	0.1837	0.1	China	0.01837		
Pickles	0.0103					
Fruits	0.0457	0.001	STMR	0.0000457		
Nut	0.0039					
Livestock and poultry	0.0795					
Milk and its products	0.0263					
Egg and its products	0.0236					
Fish and shrimp	0.0301					
Vegetable oil	0.0327	0.02	China	0.000654		
Animal oil	0.0087					
Sugar and starch	0.0044					
Salt	0.012	0.5	China	0.006		
Soy sauce	0.009					
Total	1.0286			0.0409827	0.0315	130.1

Note: ADI is the acceptable daily intake (mg/kg/d), FI (kg/day) is the dietary reference intake for a certain kind of food, NEDI is the National Estimated Daily Intake (mg), STMR (mg/kg) is the supervised trials median residues.

## Data Availability

Not applicable.

## Sample Availability

Samples are not available from the authors.
